# Internal Hernia Masquerading As Necrotizing Enterocolitis

**DOI:** 10.3389/fped.2017.00225

**Published:** 2017-10-31

**Authors:** Ranjit I. Kylat

**Affiliations:** ^1^Department of Pediatrics, College of Medicine, University of Arizona, Tucson, AZ, United States

**Keywords:** necrotizing enterocolitis, internal hernias, extreme preterm infant, acute abdomen, exploratory laparotomy

## Abstract

In extremely preterm infants, acute abdominal emergencies are fortunately less common with improving care. Spontaneous intestinal perforation and necrotizing enterocolitis are conditions where emergency surgery is most often needed. Conservative medical management and placement of temporary drain are often used in the initial management. Internal hernia (IH) is an uncommon cause of bowel obstruction in neonates, is difficult to diagnose and unfortunately are found only at autopsy. The presentation in preterm infants, distinction between these conditions, and the need for early diagnosis of IH are discussed.

## Introduction

Internal hernia (IH) is an intra-abdominal herniation of viscera through a normal or abnormal aperture within the peritoneal cavity (1). IH is an uncommon cause of intestinal obstruction or acute abdomen in children (2). It is rare to make an accurate diagnosis sufficiently early in extreme preterm infants (3, 4). The challenges in diagnosis and management of IH in a 24 weeks’ gestation preterm infant are discussed.

## Background-Case Summary

**Figure1:**
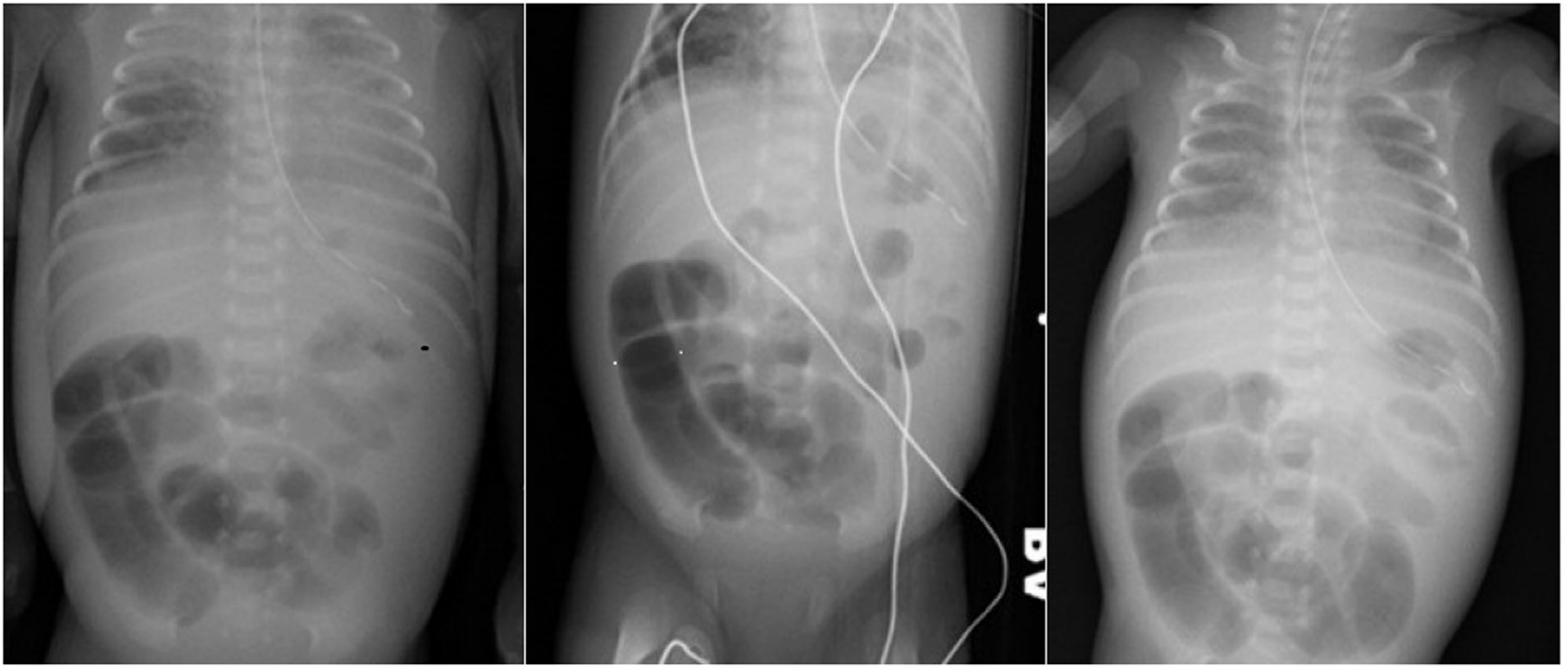
Left pane—plain AP radiograph of chest and abdomen with dilated loops of bowel. Central pane—plain left lateral decubitus radiograph of abdomen with dilated bowel loops and no evidence of pneumoperitoneum. Right pane—sequential AP radiograph of abdomen showing fixed dilated “sentinel” loops.

A 35-year-old woman with no previous medical history with regular prenatal care and normal screening tests was admitted at 23 weeks’ gestation due to premature rupture of membranes. She received betamethasone, antibiotics, and magnesium but 5 days later she went into labor and underwent emergency caesarian section for fetal bradycardia and suspected cord prolapse. A female infant was delivered at 24 weeks’ gestation with a birth weight of 625 g and required mechanical ventilation for 3 days and subsequently non-invasive ventilation. Trophic feeds and parenteral nutrition were initiated within 24 h of life. At 17 days of age, when she was tolerating close to her goal volume of feeds, she started having increasing abdominal distension and feed residuals (aspirates). Serial abdominal radiographs showed bowel dilation, which appeared fixed without evidence of pneumatosis or free intraperitoneal air (Figure 1). She had gastric decompression, broad-spectrum antibiotics, parenteral nutrition, and mechanical ventilation due to respiratory decompensation. Over the ensuing 2 days, the abdomen was more distended, firm, and started getting a darker hue. Her ventilator requirements increased and she needed high-frequency oscillator, management of profound acidosis, hyponatremia, and thrombocytopenia. Serial abdominal films did not show pneumoperitoneum or air in the portal venous system. She had raised C-reactive protein and persistent thrombocytopenia. At that point, emergency exploratory laparotomy identified a trans-mesenteric IH with a segment of gangrenous bowel. The IH was reduced and the ischemic bowel, which was mostly jejuno-ileal, was completely resected and a proximal ostomy was created. The remaining small bowel measured 35 cm from the ligament of Treitz and a viable short segment of terminal ileum with intact ileocecal valve was present. She received mechanical ventilation for 5 days, broad-spectrum antibiotics, packed red cell, and platelet transfusions. Subsequently, she was weaned off parenteral nutrition with increasing enteral feeds but did develop mild cholestasis and had her takedown of ostomy and re-anastomosis 10 weeks later. She had normal cranial ultrasound (US) scans. At 40 weeks corrected gestational age, the patient was discharged home with weight of 3.205 kg. At follow up, at 2 years of age, she had mild deficits in her receptive and expressive areas of speech on a Bailey III neuro-developmental assessment. Her growth parameters showed her to be optimal and had no evidence of gastrointestinal symptomatology. The institutional research and ethics board provided a waiver of their review but a written informed consent was obtained from the parent for publication of this report.

## Discussion

Acute abdominal emergencies needing surgical intervention are relatively uncommon with improving care in the extreme preterm infant. Among the causes, spontaneous intestinal perforation (SIP) and necrotizing enterocolitis (NEC) are by far the most common (5, 6). IH is a rare cause of intestinal obstruction and occurs at any age (1, 2). It can be congenital or acquired in origin and based on the location can be classified as trans-mesenteric, paraduodenal, paracecal/pericecal (foramen of Winslow), pelvic, and intersigmoid (mesosigmoid) hernias (1). In neonates and infants, 85% of those with IH had congenital trans-mesenteric IH (TMIH) whereas in older children and adults paraduodenal hernias are seen more often but account for less than 50% of cases. Even with contrast radiological studies, diagnosis of TMIH in infants is difficult and the value of contrast computed tomography (CT) is still unknown in this population (1).

The initial clinical course of the infant described above, with feed intolerance, abdominal distension, and dilated bowel loops could be attributed to mechanical obstruction especially in the first week of life and the differential diagnosis could include atresias, sepsis-related ileus, meconium plug or ileus or SIP. Events like apnea and bradycardia, hemodynamic instability with metabolic acidosis, disseminated intravascular coagulopathy with tense, firm, and discolored abdomen is usually assumed to worsening NEC or sepsis, especially after the first couple of weeks of age, when infants have established feeds. Initially, bowel rest, decompression, and broad-spectrum antibiotics are often instituted. Surgical consultation and intervention are more frequently done in the former group with early signs of obstruction and in the latter group is generally reserved for the patients with pneumoperitoneum and other signs of severe NEC or if there is a definite evidence of mechanical obstruction. The surgical management of a critically ill extremely preterm infant with diagnosis of severe NEC would be placement of a peritoneal (Penrose) drain, as there is a general reluctance to embark on a laparotomy in unstable infants. Systematic reviews, analyzing randomized controlled trials have not been able to answer, whether peritoneal drainage or laparotomy as treatment for perforated NEC, affects mortality or long-term sequelae or neuro-developmental outcomes (5, 7). Neither does it answer if primary anastomosis at laparotomy versus enterostomy as treatment for NEC affects mortality or long-term sequelae (5). The presence of pneumatosis on abdominal radiography may help in establishing the diagnosis of NEC. In the first few days of life, true mechanical obstruction even though uncommon, may prompt the surgeon to explore given the rare occurrence of atresia, even in the extreme premature population. But in patients who have previously been tolerating feeds, not many diagnoses, apart from NEC and rarely malrotation is entertained. In most of the reports of IH in premature infants, the onset of symptoms was early, in the first few days of life, but other reports of IH, even if the origin is congenital, can present at any age (1, 3). In a radiological review, the oldest age at presentation with IH in premature population previously reported was 24 days, but the gestational age of the patient was not known (1). Our patient, described above presenting at 17 days of age is one of very few reported patients in premature population and the only surviving one at 24 weeks’ gestation. The patient presented with non-specific signs of mechanical bowel obstruction at which point it was assumed that it was early sepsis or NEC. If an USS was performed at that time, we are not certain if the radiologist would have categorically diagnosed IH but could have provided useful information to the clinician and surgeon in revising the initial clinical diagnosis. The patient soon progressed to peritonitis and systemic inflammatory response syndrome, with thrombocytopenia and raised acute phase reactants, which was assumed to a perforated NEC. In reality, the bowel obstruction of IH progressed to gangrenous perforation resulting in laparotomy.

Sonography is being used as a complementary diagnostic modality to abdominal radiographs in infants with NEC, SIP, and malrotation (8–10). Multiple sonographic characteristics including changes in the bowel wall (thickening or thinning), perfusion, peristalsis, signs of free fluid, and air can be seen for NEC and is also useful in the sequential monitoring of these infants (8–10). In addition, it is useful for evaluation of preterm neonates with malrotation and those with SIP if they have a gasless abdomen (11, 12). Yet, an international survey of pediatric surgeons found that only 50% would utilize US as a modality for diagnosing or for following patients with NEC (13). But there are, as yet, no reports of its use in diagnosing IH in preterm infants.

Hirata has previously described two preterm infants who both died due IH and the authors lamented that “the delay in diagnosis of organic intestinal obstruction leads to fatal outcome in VLBW (preterm) infants” (14). Preterm infants often have transient feed intolerance and sometimes this can be associated with sepsis. These patients can have transient radiological signs and inflammatory markers and it would be difficult to justify the risks of undertaking laparotomy without establishing a diagnosis. Critically ill infants are unable to tolerate transportation for contrast X-ray or CT. In instances where there is suspicion of early NEC or mechanical obstruction, sequential US studies can be done as it is portable and infants are not subjected to more frequent and repeated radiation. In those unstable infants with suspected ileus or early NEC, it may be helpful in detecting the rare causes of bowel obstruction like malrotation, atresia, and IH. It is possible that if an early diagnosis of IH was made for the infant described above, either with the use of sonography or early laparotomy, she may not have needed a small segment bowel resection.

## Conclusion

In extreme low-birth weight infants with signs of feed intolerance, abdominal distension, or those with signs of NEC, US may be a useful modality for initial and sequential assessment of bowel health. In those with worsening clinical signs (as in suspected NEC in the absence of pneumatosis), it may be reasonable to embark on an exploratory laparotomy rather than continued conservative management or peritoneal drainage, given that there have been marked improvements in perioperative care, especially as conditions like IH or malrotation could be missed.

## Ethics Statement

This single case study with no identifying details was given a waiver by the institutional review and ethics board.

## Author Contributions

RK designed study, collected data and wrote the manuscript.

## Conflict of Interest Statement

The author declares that the research was conducted in the absence of any commercial or financial relationships that could be construed as a potential conflict of interest.
